# Periodontal Ligament Cell Sheets and RGD-Modified Chitosan Improved Regeneration in the Horizontal Periodontal Defect Model

**DOI:** 10.1055/s-0040-1709955

**Published:** 2020-05-12

**Authors:** Lisa R. Amir, Yuniarti Soeroso, Dewi Fatma, Hari Sunarto, Benso Sulijaya, Erik Idrus, Herlis Rahdewati, Angelia M. Tjokrovonco, Kenji Izumi, Basril Abbas, Fourier D. E. Latief

**Affiliations:** 1 Department of Oral Biology, Faculty of Dentistry, Universitas Indonesia, Jakarta, Indonesia; 2 Department of Periodontology, Faculty of Dentistry, Universitas Indonesia, Jakarta, Indonesia; 3 Division of Periodontology, Department of Oral Biological Science, Faculty of Dentistry, Niigata University, Niigata, Japan; 4 Periodontology Residency Program, Faculty of Dentistry, Universitas Indonesia, Jakarta, Indonesia; 5 Division of Biomimetics, Niigata University Graduate School of Medical and Dental Sciences, Niigata, Japan; 6 Tissue Bank, Indonesia National Atomic Energy (BATAN), Indonesia; 7 Physics of Complex Systems, Faculty of Mathematics and Natural Sciences, Bandung Institute of Technology, Bandung, Indonesia

**Keywords:** periodontal cell sheet, chitosan, arginine-glycyl-aspartic acid, horizontal periodontal defect, periodontal tissue regeneration

## Abstract

**Objective** The aim of this study was to examine the potential of periodontal ligament (PDL) cells sheet and arginine-glycyl-aspartic acid (RGD)-modified chitosan scaffold for periodontal tissue regeneration in horizontal periodontal defect model.

**Materials and Methods** PDL cell cytotoxicity was tested with 3–[4,5- dimethylthiazol-2yl]–2,5-diphenyl-2H-tetrazolium bromide assay. Cell migration toward the chitosan-based materials was analyzed with trans-well migration assay. Horizontal periodontal defect model was created in four maxillary and mandibular lateral incisors of *Macaque nemestrina* . Following periodontal therapy, the sites were transplanted with various regenerative materials: (1) chitosan, (2) RGD-modified chitosan, (3) PDL cell sheet with chitosan, (4) PDL cell sheet with RGD-modified chitosan. The periodontal tissue regeneration was evaluated clinically and radiographically. Gingival crevicular fluids were collected each week to evaluate cementum protein-1 (CEMP-1) expression with enzyme-linked immunosorbent assay, while the biopsies were retrieved after 4 weeks for histological and microcomputed tomography evaluation.

**Statistical Analysis** Data was statistically analyzed using GraphPad Prism 6 for MacOS X. Normality was tested using the Shapiro–Wilk normality test. The Kruskal–Wallis test was used to compare the groups. Significance was accepted when *p* < 0.05.

**Results** Clinical examination revealed more epithelial attachment was formed in the group with PDL cell sheet with RGD-modified chitosan. Similarly, digital subtraction radiography analysis showed higher gray scale, an indication of higher alveolar bone density surrounded the transplanted area, as well as higher CEMP-1 protein expression in this group. The incorporation of RGD peptide to chitosan scaffold in the group with or without PDL cells sheet reduced the distance of cement–enamel junction to the alveolar bone crest; hence, more periodontal tissue formed.

**Conclusions** Horizontal periodontal defect model could be successfully created in *M. nemestrina* model. Combination of PDL cell sheet and RGD-modified chitosan resulted in the higher potential for periodontal tissue regeneration. The results of this study highlight the PDL cell sheet and RGD-modified chitosan as a promising approach for future clinical use in periodontal regeneration.

## Introduction

Periodontal defects are frequently found in severe periodon-titis patients and various attempts have already been performed to regenerate the new periodontal tissues. 1 2 3 The current treatments provide predictable clinical outcome of the improvement in clinical parameters; however, these approaches are restricted in appropriate case selection of small-to-medium-size defect. In critical size defect such as in one-wall or horizontal periodontal defect, periodontal tissue regeneration is still a challenge and it often results in unpredictable clinical outcome. As such focus has shifted to the potential use of tissue engineering technique for periodontal regeneration in large bone defect cases. 4 5 6 7 8 9

The application of cell sheet for periodontal regenerative therapy has been recently reported. 6 7 8 9 Thermoresponsive surfaces such as poly(N-isopropylacrylamide/ PIPAAm) give the possibility for nonenzymatic treatment to harvest the cells, thereby protecting the cell junction, cell surface proteins and extracellular matrix (ECM) proteins. Cells from various sources such as periodontal ligament (PDL), bone marrow, and adipose tissues have been tested for the synthesis of cell sheet materials. 6 7 8 9 Although the initial concept of cell sheet technology is to eliminate the need for scaffold materials, due to delicate nature of the cell sheet, the use of biodegradable support matrices is still necessary to support the cell sheet to be transplanted to the defect areas such as periodontal tissue defects.

One of the promising biomaterials to be used for tissue regeneration is the naturally occurring polymer of chitosan. 10 11 12 13 14 Chitosan is a partially deacetylated form of chitin, a polysaccharide present in the exoskeleton of crustaceans shells. Its characteristics include biocompatible, biodegradable, bioactive, and versatility in surface chemistry; all of these features make chitosan an attractive scaffold material for tissue engineering purposes. We previously showed that in addition to chitosan’s role as a three-dimensional scaffold for osteogenic cells, chitosan has the capacity to stimulate dental pulp stromal cells (DPSCs) proliferation and early osteogenic differentiation *in vitro* comparable to the well-known osteogenic supplement of dexamethasone. 14 Tripeptide arginine-glycine-aspartic acid (RGD) motif presents in various adhesive proteins in the ECM and is a well-known general cell recognition motif via the cell surface integrin receptors. The incorporation of RGD peptide to the scaffold biomaterials was reported to improve the cell attachment to the biomaterials. 15 16

Despite several studies demonstrated chitosan biomaterials in combination with RGD peptide as a promising scaffold material for bone and cartilage tissue engineering, 17 18 19 20 no study reported the potential of RGD-modified chitosan to induce periodontal regeneration in horizontal periodontal defect cases. It is currently unknown whether the combination of periodontal cell sheet and RGD-modified chitosan could improve the formation of periodontal tissue particularly in horizontal periodontal defect case. We hypothesized that the addition of RGD in the chitosan scaffold could improve the periodontal tissue regeneration capacity of PDL cell sheet. The aim of this study was to examine the effect of PDL cell sheet and RGD-modified chitosan construct in stimulating periodontal tissue regeneration of horizontal periodontal defect in *Macaque nemestrina* model.

## Materials and Methods

### Materials

α- Minimum Essential Medium (MEM) fetal bovine serum, penicillin, streptomycin, fungizone, and collagenase I were all from Gibco (Life Technologies; Grand Islands, NY, USA). RGD peptide, ascorbic acid, β-glycerophosphate, dexamethasone, and 3–[4,5- dimethylthiazol-2yl]–2,5-diphenyl-2H-tetrazolium bromide (MTT) powder were purchased from Sigma (St. Louis, MO, United States). Dispase was from Roche (Indianapolis, United States). All culture plates were and transwell polyethylene terephthalate (PET) membrane were form Costar (Corning, New York, United States). Tubes were from BD Falcon (New Jersey, United States). UpCell dish were from Nunc; ThermoFisher Scientific, United States. Bradford Protein Assay was from Bio-Rad protein assay kit (Bio-Rad, United States). Human cementum protein 1 (CEMP-1) enzyme-linked immunosorbent assay kit was from Cusabio (Wuhan, China).

### RGD-Modified Chitosan Preparation

Chitosan-based materials were prepared at the Center for Application of Isotope and Radiation Technology, Indonesia National Atomic Energy Agency. 14 Chitosan with the degree of deacetylation of 94.5% was dissolved in 1% v/v acetic acid (0.1 M) and stirred until fully dissolved to obtain a homogenous 2 wt% chitosan solution. RGD-modified chitosan scaffold was prepared by physical adsorption of RGD peptide to chitosan. Four milligrams of RGD peptide were added to 50 mL chitosan solution, casted to a custom-made mold, and freezed for 24 hours. The RGD-modified scaffold was then solidified in 1M NaOH/ethanol solution, neutralized in distilled water and freeze-dried. Scaffold porosity was ~175 μm. 21

### Cell Culture

This protocol received an ethical clearance from Primate Research Center, Bogor Institute of Agriculture’s Animal Care and Use Committee (ACUC No IPB PRC-15-B0012) and followed the Animal Research: Reporting of In Vivo Experiments (ARRIVE) guidelines. PDLs were collected from four *Macaque nemestrina* upper first incisors. Cell isolation procedures were as previously described. 14 22 Briefly, PDL tissue was obtained from apical two-thirds of the root. Enzymatic dissociation was performed with 4 mg/mL collagenase I and 3 mg/mL dispase in a 15 mL tube at 37°C under 5% CO _2_ in an incubator for 1 hour. Cells were then plated into six-well plate and cultured with basal medium consisted of α-MEM, 10% Fetal Bovine Serum (FBS), 100 U/mL penicillin, 100 μg/mL streptomycin, and 1.25 μg/mL fungizone until they reached ~90% confluency for ~14 days. Total heterogenous PDL cell population from passage 1 to 4 was used for experiments. Cell cytotoxicity analysis was performed at 24 hours following incubation of PDL cells and chitosan-based scaffold with MTT assay as previously described. 14 The ability of chitosan scaffold to induce cell migration was tested using 8.0 μM Transwell PET membrane. 5.10 5 cells were seeded in the upper compartment of the well insert, while the chitosan scaffold was placed in the lower compartment and incubated for 4 hours. The migrated cells in the lower compartment were counted in the microplate reader (Benchmark, Bio-Rad) at 655 nm. Data were corrected for blank values (medium only). The experiments were repeated twice and were performed in triplicate. PDL cell sheets were obtained cultured in 10 mm UpCell dish for 3 weeks in osteogenic medium containing basal medium supplemented with 100 μg ascorbic acid, and 10 mM β-glycerophosphate and 10 nM dexamethasone. Prior to transplantation, PDL cell sheets were harvested, loaded onto the chitosan-based membrane and the cell-scaffold constructs were incubated in 37°C to facilitate cell attachment.

### Surgical Procedures

**Fig. 1:**
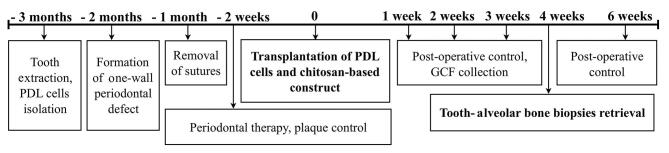
Research timeline. GCF, gingival crevicular fluid; PDL, periodontal ligament.

Four adults (6–8 years old) male *Macaque nemestrina* (weight 15–18 kg) were considered in this study. All procedures were performed under general anesthesia using isopropyl IV (12 mg/kg body mass index) and local anesthesia (Xylocaine-adrenalin 5 mg/mL). Horizontal periodontal defect was initially created on four maxillary and mandibular lateral incisors using orthodontic elastic bands in the sulcus areas. 23 However, the concavity of *Macaque nemestrina* tooth morphology prevented the elastic bands to stay in place; the surgical approach was later introduced using 1.5 mm rounded-end burr to remove the surrounding alveolar bone in 16 lateral incisors. Horizontal periodontal defect defined as a 5 × 3 mm (height x width) of horizontal bone loss with reference point of cement–enamel junction. 24 25 All animals were tolerated well with the procedures. Periodontal therapy was introduced 6 weeks after periodontal defect creation that consisted of plaque control and topical irrigation with 0.2% chlorhexidine digluconate and minocycline HCl solution. Two weeks following periodontal therapy, lateral incisor sites were divided into four groups based on the regenerative materials: (1) chitosan as control group, (2) RGD-modified chitosan, (3) PDL cell sheet seeded in chitosan membrane group, and (4) PDL cell sheet seeded in RGD-modified chitosan membrane group. Regenerative materials were securely placed on root surfaces and their respective alveolar bone, and the flap was then secured with 5–0 nylon sutures. Clinical periodontal parameters measurement included pocket depth, clinical attachment loss (CAL), and bleeding on probing. An increase in epithelial attachment was determined by subtracting CAL at 4 weeks from CAL before cell-scaffold constructs transplantation. Gingival crevicular fluids (GCFs) were collected every week up to 4 weeks. Biopsies consisted of lateral incisor teeth and surrounding alveolar bone were taken 4 weeks after transplantation of the regenerative materials. The sites were then filled with the carbonite apatite graft (GAMA-CHA; Jogjakarta, Indonesia). This was a survival study, whereby all animals remained in the facility following biopsy retrieval. In 6-month follow-up, all animals behaved and grew well. Research timeline was described in Fig. 1 . Following micro-computed tomography (micro-CT) examination, the biopsies were embedded in paraffin, sectioned with 5 µM thickness and stained with hematoxylin and eosin for histological analysis.

### Radiographic Examination

Digital parallel periapical radiograph was performed to measure the bone height using cone indicator and bite registration. X-ray unit (Rextar X, 70 kV/2 mA) was used with a 0.12 second exposure time for the anterior teeth before transplantation and each week up to 4 weeks following transplantation. For the comparison purpose, the contrast was adjusted on both radiographic images before and 4 weeks after treatment, using digital subtraction radiography program, from MatLab image registration software. Both radiograph images were overlapped and registered to obtain any density differences. The region of interest (ROI) was 30 × 30 pixels at the top of alveolar bone whereby the regenerative materials were transplanted. The program was then processed the average value of the gray scale within the ROI.

### CEMP-1 Protein Expression

GCFs were collected every week up to 4 weeks prior to biopsies retrieval. GCF total protein was measured with Bradford Protein Assay and subsequently standardized up to 200 µg/mL. CEMP-1 protein expression was measured according to manufacturer’s suggested protocols (Cusabio).

### Micro-CT Examination

Biopsies consisted of lateral incisor teeth and surrounding alveolar bone was scanned using SkyScan 1173 (Bruker-Micro-CT; Kontich, Belgium) at a voltage of 55 kV, a current of 145 μA, an integration time of 600 milliseconds, a resolution of 12.11 μm, and a rotation step of 0.2 degrees. A series of projection images in a 16-bit TIFF format was obtained from the scanning process and further reconstructed using NRecon 1.7.3.1 (Bruker-Micro-CT) using the GPUReconServer. The scanning was followed by a reconstruction using NRecon 1.7.3.1 (Bruker-Micro-CT) with the GPUReconServer. Basic image processing and qualitative and quantitative analyses were done using DataViewer, CTAn (Bruker-Micro-CT) and ImageJ 1.45r (National Institute of Health; Bethesda, Maryland, United States).

### Statistical Analysis

Data was statistically analyzed using GraphPad Prism 6 for MacOS X. Normality was tested using the Shapiro–Wilk normality test. Data were analyzed using the Kruskal–Wallis test, and significance was accepted when *p* < 0.05.

## Results

**Fig. 2:**
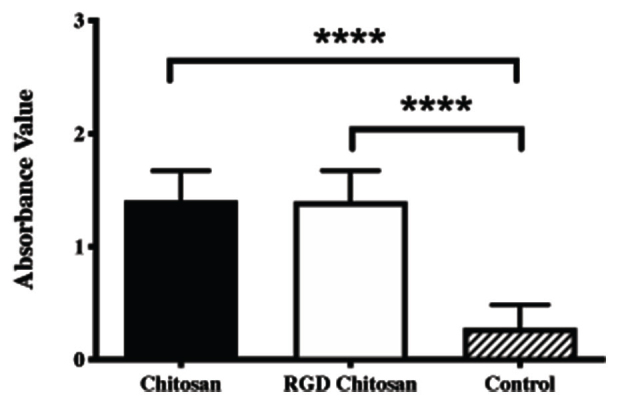
Cytotoxicity assay. RGD, arginine-glycyl-aspartic acid.****p<0.0001.

**Fig. 3:**
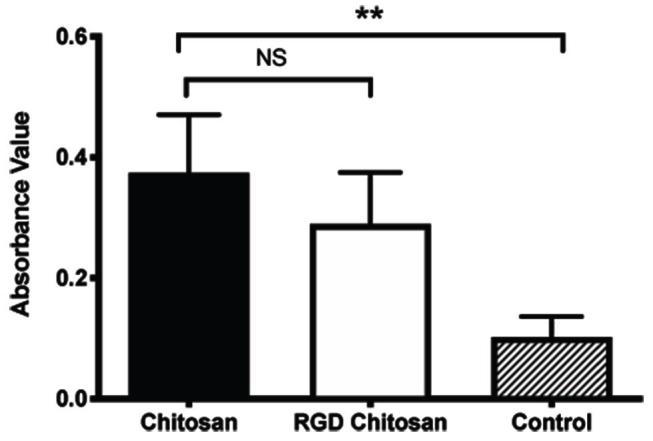
PDL cell migration. PDL, periodontal ligament; RGD, arginine-glycyl-aspartic acid. NS, not significant.**p<0.0001.

**Fig. 4:**
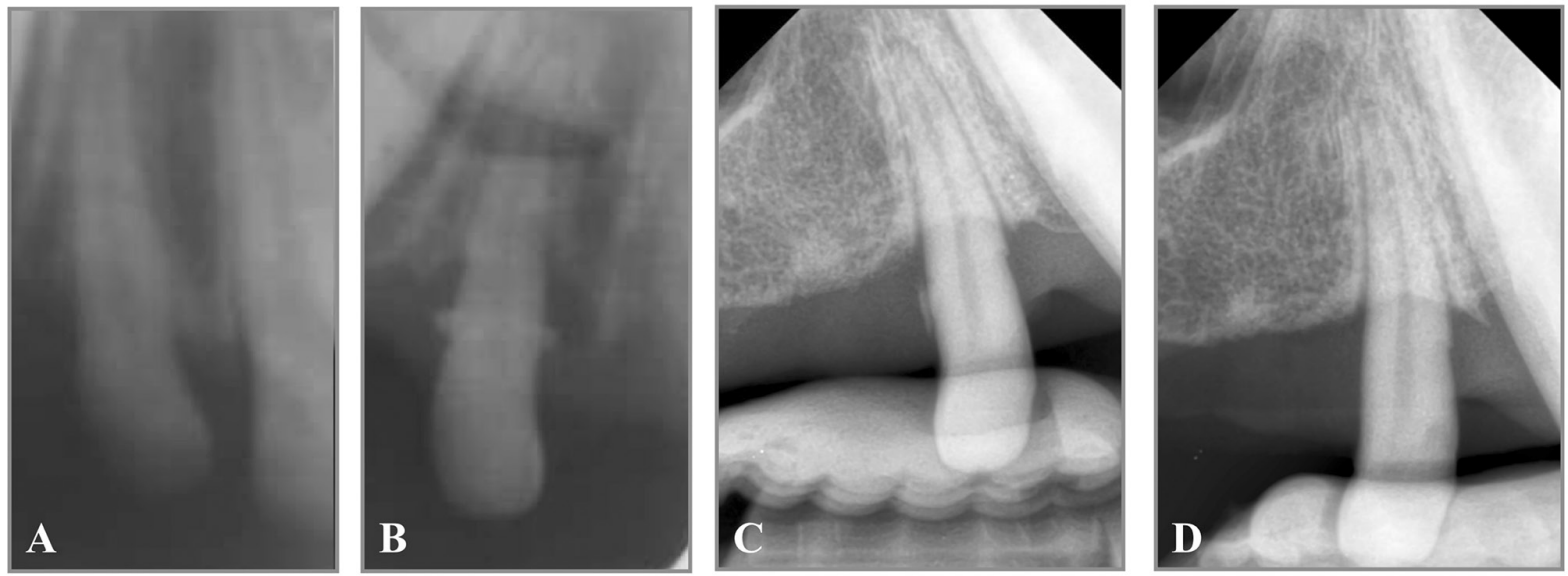
Radiographic analysis of the alveolar bone. ( **A** ) Baseline. ( **B** ) 1 week after surgical bone defect creation. ( **C** ) Before cell-scaffold construct transplantation. ( **D** ) 4 weeks after periodontal regenerative therapy.

**Fig. 5:**
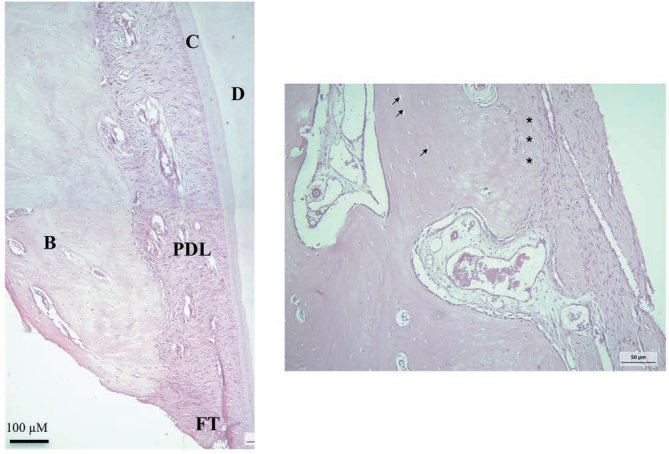
( **A** ) Micrograph of the regenerated periodontal tissue. B, bone; C, cementum; D, dentin; FT, fibrous tissue; PDL, periodontal ligament. ( **B** ) Exiting alveolar bone. Note that flattened osteocytes resided in the existing alveolar bone (arrows) adjacent to the newly regenerated bone (asterisks).

**Fig. 6:**
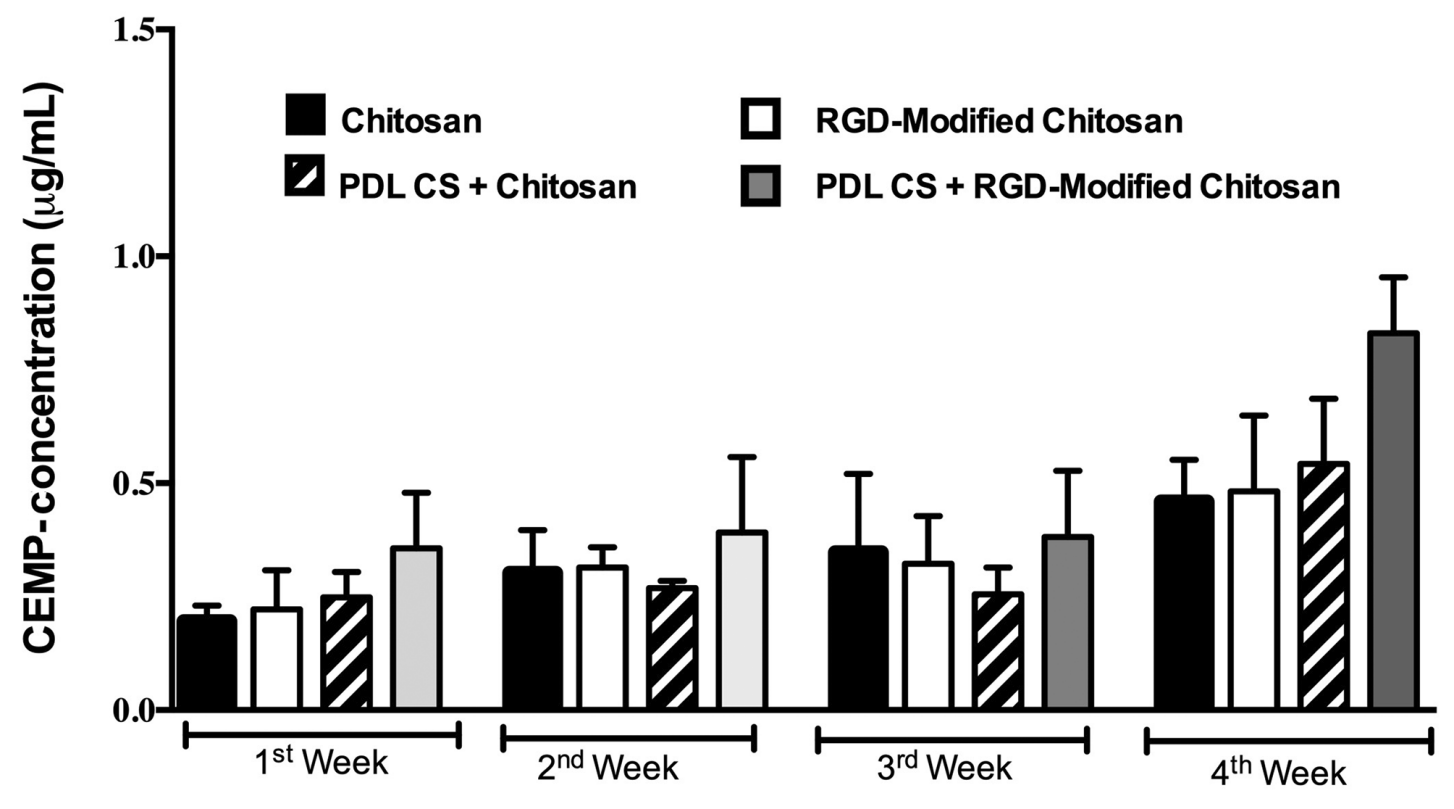
CEMP-1 expression. CEMP-1, cementum protein-1; PDL, periodontal ligament; RGD, arginine-glycyl-aspartic acid.

**Fig. 7:**
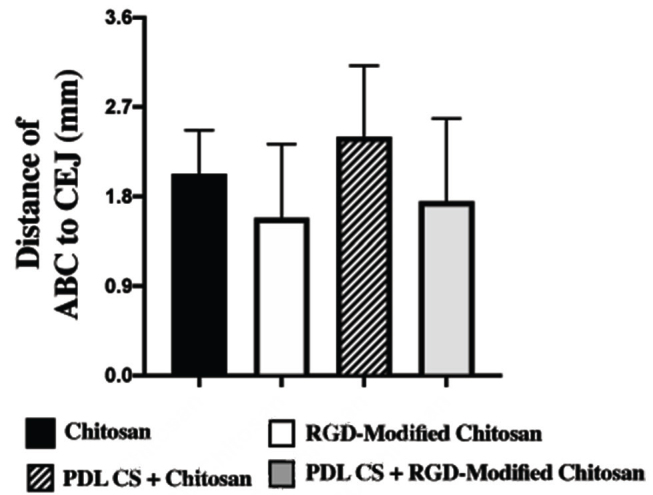
Microcomputed tomography scan analysis of distance of alveolar bone crest (ABC) to cement–enamel junction (CEJ). The shortest distance of alveolar bone crest and cement–enamel junction indicated more newly formed periodontal tissue. PDL, periodontal ligament; RGD, arginine-glycyl-aspartic acid.

We first tested the biomaterials *in vitro* . MTT assay showed biocompatibility of chitosan and RGD-modified chitosan ( Fig. 2 ). Higher cell proliferation was observed in both biomaterials tested ( *p* < 0.0001). More than 50% of the seeded cells were migrated toward lower compartments in the Boyden chamber in both chitosan and RGD-modified chitosan group ( *p* < 0.01) ( Fig. 3 ). The initial alveolar bone height before the *in vivo* experiments was presented in Figs. 4A and served as baseline. Horizontal periodontal defect was successfully created in the lateral incisors with the average loss of attachment of 5.45 ± 0.33 mm ( Fig. 4B 4C ). No significant different in the loss of attachment of lateral incisors was found in all macaques. Four weeks following PDL cell sheet and chitosan-based scaffold construct transplantation to the periodontal horizontal defect, an increase in epithelial attachment was measured ( Table 1 ). The clinical attachment level gain was observed in all group tested. In the group treated with PDL cell sheet with RGD-modified chitosan, 3 mm increase in epithelial attachment was observed. The increase was 1.7-fold higher than the group treated with chitosan. The epithelial attachment gain reached 55% of the initial attachment prior to horizontal periodontal defect formation as measured clinically. Alveolar bone density was evaluated at week 4 and was shown in Table 2 Fig. 4D . Histological analysis showed the regenerated periodontal tissues in the defect areas, while the most coronal part was still filled with fibrous tissue ( Fig. 5A ). The regenerated alveolar bone indicated by the presence of young osteocytes, characterized by their round shaped and abundant cytoplasm due to higher metabolic activity ( Fig. 5B ). The existing bone was seen in a more apical part of alveolar bone indicated with the presence of mature osteocytes with a more flattened shaped and with cement lines marking the newly deposited bone ( Fig. 5B ). CEMP-1 protein expression consistently increased over period of time in all group tested ( Fig. 6 ). The highest CEMP-1 expression was observed in the group treated with PDL cell sheet and RGD-modified chitosan. In line with this finding, the shortest distance of alveolar bone crest and cement–enamel junction was observed in PDL cell sheet and RGD-modified chitosan ( Figs. 7 8 ).

**Table 1:** The average increase in clinical epithelial attachment

Group	Increase in epithelial attachment (mm) (Mean ± SD)
Chitosan	1.75 ± 0.71
RGD-modified chitosan	2.13 ± 0.83
PDL cell sheet chitosan	2.25 ± 0.71
PDL cell sheet RGD-modified chitosan	3.00 ± 0.75
Abbreviations: PDL, periodontal ligament; RGD, arginine-glycyl-aspartic acid; SD, standard deviation.	

**Table 2:** The average gray scale on alveolar bone density subtraction results

Group	Gray scale of alveolar bone density (Mean ± SD)
Chitosan	7.31 ± 10.27
RGD-modified chitosan	16.70 ± 13.17
PDL cell sheet chitosan	19.34 ± 21.46
PDL cell sheet RGD-modified chitosan	21.98 ± 7.85
Abbreviations: PDL, periodontal ligament; RGD, arginine-glycyl-aspartic acid; SD, standard deviation.	

**Fig. 8:**
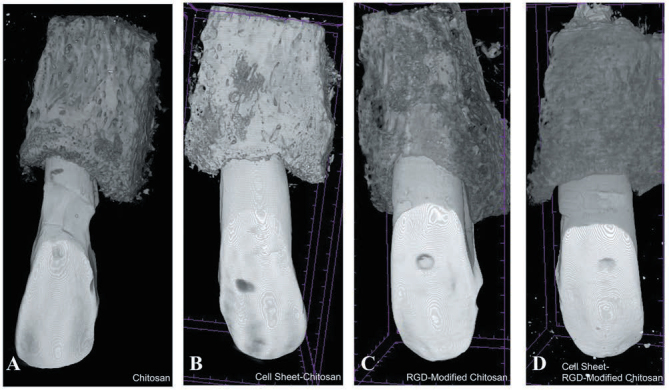
Micro-computed tomography scan analysis. ( **A** ) Chitosan. ( **B** ) Cell sheet-chitosan. ( **C** ) RGD-modified chitosan. ( **D** ) Cell sheet-RGD modified chitosan. RGD, arginine-glycyl-aspartic acid.

## Discussion

The present study evaluated the potential of PDL cell sheet and chitosan-based materials for periodontal tissue regeneration in the periodontal horizontal defect in *M. nemestrina* model. The oral conditions of *M. nemestrina* share many similarities with humans as well as the healing process that resembles healing process in humans. 26 The periodontal horizontal defect was successfully created in the second incisors by surgical approach to remove the alveolar bone surrounding the roots and in combination with the application of elastic bands in the cervical area to induce plaque accumulation that resembles a more natural periodontal tissue destruction process. 23 Previously, we have reported the biocompatibility and osteoconductivity properties of chitosan in DPSCs and PDL cells. 14 The chitosan scaffold was able to stimulate the proliferation activity of these cells. We also found that PDL cells did not express mesenchymal stromal cells (MSCs) markers; still they have the capacity to differentiate toward osteoblastic lineage comparable to PDL cells with MSCs markers (CD73, CD90 and CD105) (unpublished data). Therefore, in this study we induced the heterogenous PDL cells with osteogenic supplements for the formation of cell sheet.

Four weeks following regenerative therapy, the clinical attachment level gain was detected in all group tested. PDL cell sheet seeded in RGD-modified chitosan showed more clinical attachment gain; more than 50% compared with the level before regenerative therapy was introduced. The data was consistent with the micro-CT analysis that revealed the shortest distance between cement–enamel junction to alveolar bone crest; an evidence for a more periodontal tissue formation was seen in the group treated with PDL cell sheet and RGD-modified chitosan. The newly formed periodontal tissue attachment was further analyzed by the expression of CEMP-1, the key regulator of cementogenesis. CEMP-1 protein expression was consistently increased over the period of the periodontal tissue regeneration process. CEMP-1 was synthesized by cementoblasts and their progenitors in the PDL and was known to promote cementoblasts attachment, differentiation as well as the hydroxyapatite crystals formation. 27 28

The goal of periodontal regenerative therapy following the elimination of the etiology of periodontitis is to achieve periodontal tissue regeneration. 29 30 31 The formation of new alveolar bone and cementum with the supportive PDLs restores the periodontal tissue to its previous form and function. The amount of existing intact bony wall will determine the regeneration process. Crater-form defect would give sufficient mechanical and biological support to the cell-tissue construct. 32 In one-wall bone defect or horizontal bone defect cases however, periodontal tissue regeneration process is a challenge, due to a minimal existing healthy bony wall, lacking of vascularization and healthy cells. 4 5 To overcome this limitation, MSCs-based tissue engineering is believed to generate a more predictable clinical outcome. 4 5 6 7 8 9 The optimal cell delivery method to the defect area is crucial to maintain cell survival. In recent years, studies demonstrated that cell sheet provides a better cell delivery method, as it can generate high-density cells with abundant endogenous ECM, protects cell–cell junction, and cell surface proteins. 6 7 8 9 Cell sheet from various sources of MSCs has been tested for periodontal tissue regeneration. 6 7 8 9 33 34 Transplantation PDL cell sheet to a periodontal tissue defect model resulted in a significant periodontal regeneration with newly formed cementum and well-oriented PDL fibers. 33 34 Due to the loose structure of cell sheet, scaffold biomaterials are still essential to support cell sheet in periodontal tissue reconstruction particularly in critical size defect.

Biomaterial scaffold provides not only the temporary structural integrity but it also needs to support its interaction with the cells. In the local environment, interaction between cells and ECM has a significant impact of on cell fate for their adhesion, proliferation, and differentiation. Mimicking this local environment would be crucial for maintaining cells and to differentiate to a distinct phenotype of cells and to form the desired tissues. Incorporating the adhesion ligands as biochemical elements in biomaterials is often performed to obtain functionalized surfaces to control cell behavior and cellular pathways. The RGD peptide sequence has been long recognized for an essential binding motif for specific transmembrane protein that is involved in cellular adhesion to the various ECM proteins. The addition of RGD peptide to the chitosan scaffold for tissue regeneration was intended to support the cells adhesion and to increase the number of infiltrated and proliferated cells and into the scaffold. 15 16 35 Various studies reported the benefit of incorporating RGD peptide on biomaterials on osteogenic differentiation. 17 18 19 20 In agreement with others, our study demonstrated the potential of RGD modified-chitosan in periodontal regeneration that reached 50% clinical attachment level gain, 4 weeks following transplantation of PDL cell sheet and RGD-modified chitosan scaffold in the horizontal periodontal defect. Although in the crestal areas of alveolar bone fibrous tissues were still apparent, these cells were immunopositive for collagen type I and osteopontin (manuscript in preparation). This indicates the osteogenic potentials of these cells that at the later time might eventually differentiate and form the new alveolar bone. The long-term fate of the newly regenerated tissues warrants further studies.

## Conclusion

In conclusion, horizontal periodontal defect model was successfully created in *M. nemestrina* model. Combination of PDL cell sheet and RGD-modified chitosan resulted in the higher potential for periodontal tissue regeneration. The results of this study highlighted the PDL cell sheet and RGD-modified chitosan as a promising approach for future clinical use in periodontal regeneration.
